# UHPLC-MS-Based Serum and Urine Metabolomics Reveals the Anti-Diabetic Mechanism of Ginsenoside Re in Type 2 Diabetic Rats

**DOI:** 10.3390/molecules26216657

**Published:** 2021-11-03

**Authors:** Heyu Wang, Yaran Teng, Shinan Li, Ying Li, Hui Li, Lili Jiao, Wei Wu

**Affiliations:** Jilin Ginseng Academy, Changchun University of Chinese Medicine, Changchun 130117, China; Heyu2006@163.com (H.W.); tyr313006979@163.com (Y.T.); lishinan0914@163.com (S.L.); ying060111@163.com (Y.L.); lihuiterrisa@163.com (H.L.); jiaoaj@hotmail.com (L.J.)

**Keywords:** ginsenoside Re, type 2 diabetes, UHPLC-MS, metabolomics

## Abstract

*Panax ginseng* was employed in the treatment of “Xiao-Ke” symptom, which nowadays known as diabetes mellitus, in traditional Chinese medicine for more than a thousand years. Ginsenoside Re was the major pharmacologic ingredient found abundantly in ginseng. However, the anti-diabetic of Ginsenoside Re and its underlying mechanism in metabolic level are still unclear. Serum and urine metabolomic method was carried out to investigate the anti-diabetic pharmacological effects and the potential mechanism of Ginsenoside Re on high-fat diet combined streptozotocin-induced type 2 diabetes mellitus (T2DM) rats based on ultra-high-performance liquid chromatography coupled with quadrupole exactive orbitrap mass spectrometry (UHPLC-Q-Exactive Orbitrap/MS). Serum and urine samples were collected from the control group (CON), T2DM group, metformin (MET) treatment group, and ginsenoside Re treatment group after intervention. The biochemical parameters of serum were firstly analyzed. The endogenous metabolites in serum and urine were detected by UHPLC-MS. The potential metabolites were screened by multivariate statistical analysis and identified by accurate mass measurement, MS/MS, and metabolite databases. The anti-diabetic-related metabolites were analyzed by KEGG metabolic pathway, and its potential mechanism was discussed. The treatment of ginsenoside Re significantly reduced the blood glucose and serum lipid level improved the oxidative stress caused by T2DM. Biochemical parameters (urea nitrogen, uric acid) showed that ginsenoside Re could improve renal function in T2DM rats. Respective 2 and 6 differential metabolites were found and identified in serum and urine of ginsenoside Re compared with T2DM group and enriched in KEGG pathway. Metabolic pathways analysis indicated that the differential metabolites related to T2DM were mainly involved in arachidonic acid metabolism, Vitamin B6, steroid hormone biosynthesis, and bile secretion metabolic pathways. This study verified the anti-diabetic and anti-oxidation effects of ginsenoside Re, elaborated that ginsenoside Re has a good regulation of the metabolic disorder in T2DM rats, which could promote insulin secretion, stimulated cannabinoid type 1 receptor (CB_1_), and CaMKK β to activate AMPK signaling pathway, inhibited insulin resistance, and improved blood glucose uptake and diabetic nephropathy, so as to play the role of anti-diabetic.

## 1. Introduction

The epidemic of diabetes mellitus (DM) and its complications poses a major threat to global health; about 1 out of 11 adults worldwide have DM, which is the ninth major cause of death globally [1]. DM is classified into type 1 diabetes mellitus (T1DM) and type 2 diabetes mellitus (T2DM), and nearly 90% of cases are T2DM, which is caused by insulin resistance and lack of proper response to hyperglycemia [2]. DM can cause serious health problems, which may damage almost all organs in the body, such as retinas, liver, and kidneys, and most T2DM patients have at least one complication [3]. It has been reported that oxidative stress was one of the causes of insulin resistance and impaired insulin secretion, which eventually leads to T2DM [4]. At the same time, continuous oxidative stress and dysfunction of various metabolic pathways were also the main cause of diabetic complications, such as diabetic nephropathy [5]. Currently, the treatment of T2DM is mainly focused on the injection of insulin or its peptide derivatives, oral anti-diabetic drugs, and dietary control. Insulin injection brings inconvenience to patients’ daily life, while long-term oral administration of chemical drugs may cause side effects. Researchers are exploring new alternative medicines for the treatment of T2DM, such as traditional herbal medicines, which were attracting increasing attention because of their less toxicity and side effects [6].

Ginseng (*Panax ginseng* C. A. Meyer) belongs to the genus *Panax* of Araliaceae, which has long been used to treat T2DM as a famous herbal medicine [2]. Ginsenosides were the main active component found abundantly in ginseng. According to its structure, it can be summarized as 3 groups, panaxadiol type (Rc, Rd, Rb_1_, Rb_2_, Rb_3_, and Rg_3_), panaxatriol type (Rh_1_, Rg_1_, Rg_2_, Rf, and Re) and oleanolic acid type (Ro) [7]. It was found that the content of ginsenoside in ginseng berry was four times higher [8] and ginsenoside Re was seven times higher [9] than that in ginseng root. Moreover, ginseng berry had stronger hypoglycemic activity than ginseng root [10].

Ginsenoside Re was one of the most abundant saponins components in ginseng [11], which has various pharmacological properties, such as improved myocardial fibrosis [7], cardio-protective effects [12], improving cardiovascular disease [13], protective and anti-angiopathy effects [14], antioxidant, anti-inflammatory, and anti-apoptotic effects [15]. The most effective aspects were related to the treatment of diabetes [14,16,17,18,19]. It was reported that ginsenoside Re had the effects of hypoglycemic, hypolipidemic, anti-oxidant stress, improving insulin resistance, increasing insulin level, and attenuating diabetes-associated cognitive decline. The possible mechanism of these effects was that ginsenoside Re could improve diabetes by activating the AMPK signaling pathway [3,18]. However, the potential metabolic mechanism of ginsenoside Re in improving diabetes has been not reported. Based on the previous studies, we found that ginseng berry had excellent anti-diabetic and antioxidant effects [20]. Ginsenoside Re, as the highest monomer component contented in ginseng berry, may play an important role in pharmacodynamic actions. Therefore, it is necessary to study the anti-diabetic effect and its potential mechanism of ginsenoside Re for screening valuable natural compounds.

With the increasing sensitivity and high throughput of metabolomics technology, the discovery of biomarkers for predicting the onset and severity of diseases has become an important diagnostic tool for clinicians [21]. UHPLC-MS-based metabolomics screening could provide important information for understanding the pathophysiological pathway of T2DM, which helps to establish effective interventions to treat type 2 diabetes [22].

In this study, serum and urine metabolomics were analyzed by UHPLC-Q-Exactive Orbitrap/MS to study the metabolic profiles and differential metabolites of ginsenoside Re in the treatment of T2DM rats. And the hypoglycemic, hypolipidemic, and anti-oxidant stress effects were verified, which aimed to discover the effects of ginsenoside Re on different metabolic pathways and provide potential mechanisms of anti-diabetic effects of ginseng and ginseng berry ginsenosides in T2DM rats.

## 2. Results

### 2.1. Anti-Diabetic Effects of Ginsenoside Re

**Figure 1:**
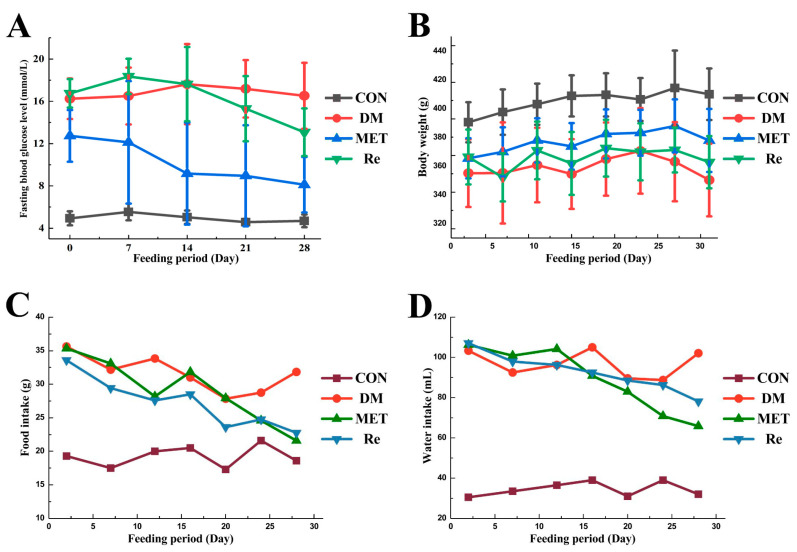
Effect of ginsenoside Re on fasting blood glucose level (**A**), body weight (**B**), food (**C**), and water (**D**) intake in T2DM rats.

The effect of ginsenoside Re on the fasting blood glucose (FBG) level, body weight (BW) gain, and food and water intake in T2DM rats are shown in Figure 1. After the establishment of the T2DM rat model, the model rats were randomly divided into T2DM (T2DM with normal saline), MET (T2DM with Metformin), and Re (T2DM with Ginsenoside Re) groups. The FGB and BW of each model rat were different, so the average values of the three groups were also different. Therefore, the starting points in Figure 1A,B were different. The FBG level of metformin and ginsenoside Re treatment group decreased gradually. There was no significant difference in the control and T2DM groups (*p* > 0.05). After 30 days of treatment, the FBG level in the MET and Re groups significantly decreased from 12.75 ± 2.37 mmol/L to 8.48 ± 2.79 mmol/L (*p* < 0.01) and 16.75 ± 1.35 mmol/L to 13.07 ± 2.25 mmol/L (*p* < 0.05), the hypoglycemic rate was 21.97%. During the 30 days of intervention, the food and water intake of MET and Re groups showed a decreasing trend, while it was no significant difference in the change of BW (*p* > 0.05), prompting that the effect of ginsenoside Re on reducing the FBG level was not due to the BW loss of the rats, but due to its pharmacological effect.

As shown in Table 1, ginsenoside Re treatment significantly reduced the level of LDL-c and TG (*p* < 0.05). However, there are no significant differences in TC (*p* = 0.07), HDL-c (*p* = 0.42), and NEFA (*p* = 0.07) among the groups, suggesting that treatment of ginsenoside Re could reduce the blood lipid level of T2DM rats. Ginsenoside Re treatment significantly reduced the level of BUN (*p* < 0.05), and the level of UA also showed a tendency to decrease, but the differences (*p* = 0.26) were not statistically significant. It is suggested that ginsenoside Re could improve the renal function of T2DM rats and has the potential to treat diabetic nephropathy. Ginsenoside Re significantly increased the activities of SOD (*p* < 0.05) and decreased the level of MDA (*p* < 0.05) in T2DM rats, and the activities of CAT also tended to increase, but the difference was not statistically significant, indicating that ginsenoside Re could mitigate the oxidative stress in T2DM rats. The purpose of determining the biochemical indexes of rats was to observe the biological effects after drug intervention, that was, whether drugs can improve and treat diseases. After determining that the drug had positive biological effects, metabolomics was used to explore the metabolic pathways involved in regulation in this process, and then, explored the potential mechanism of the drug’s pharmacological effect.

### 2.2. Multivariate Statistical Analysis Based on UHPLC-MS

**Figure 2:**
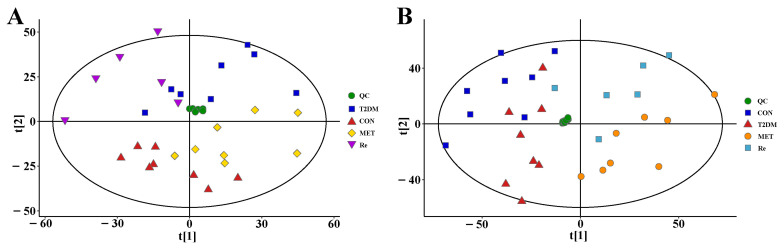
The PCA score plots of all samples from serum (**A**) and urine (**B**).

**Figure 3:**
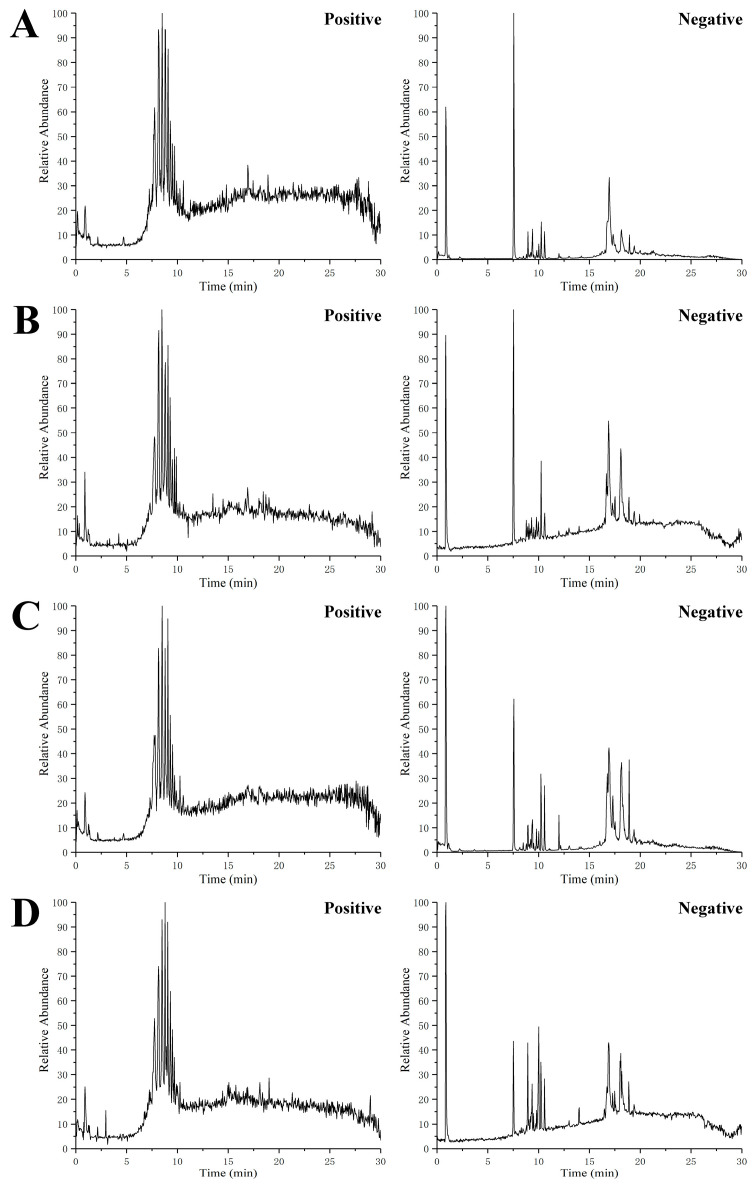
Representative UHPLC-MS total ion chromatograms (TIC) of serum from the control group (**A**), T2DM group (**B**), metformin group (**C**), and ginsenoside Re (**D**) group, respectively.

**Figure 4:**
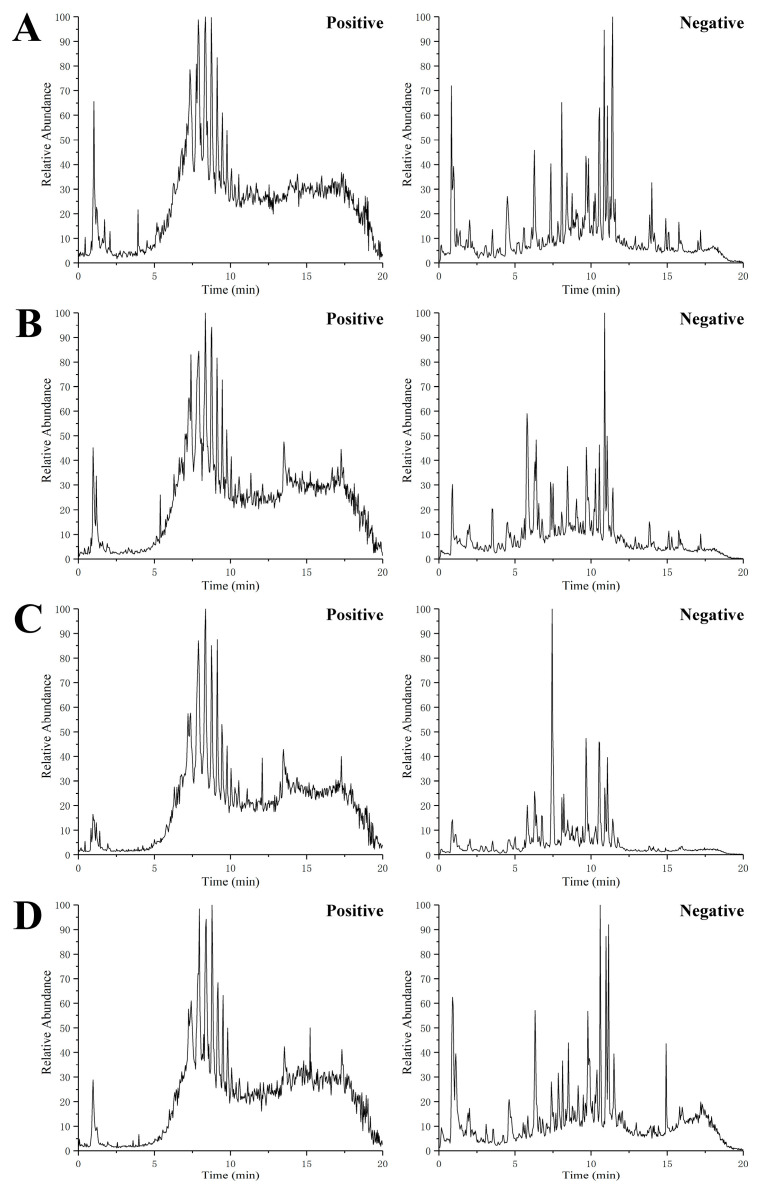
Representative UHPLC-MS total ion chromatograms (TIC) of urine from the control group (**A**), T2DM group (**B**), metformin group (**C**), and ginsenoside Re (**D**) group, respectively.

In order to ensure the reliability and stability of the system and data, QC sample was detected in every six samples over the acquisition process, and the PCA model was performed on all the samples from serum and urine, as shown in Figure 2, indicating that the method showed good stability and low variation caused by the instrumental error. Total ion chromatograms (TIC) of the serum and urine samples from CON, T2DM, MET and Re groups were shown in Figure 3 and Figure 4, respectively. Suitable chromatographic and MS conditions were selected for the measurement of the samples in this study. UHPLC-MS metabolic profiling analysis was performed.

**Figure 5:**
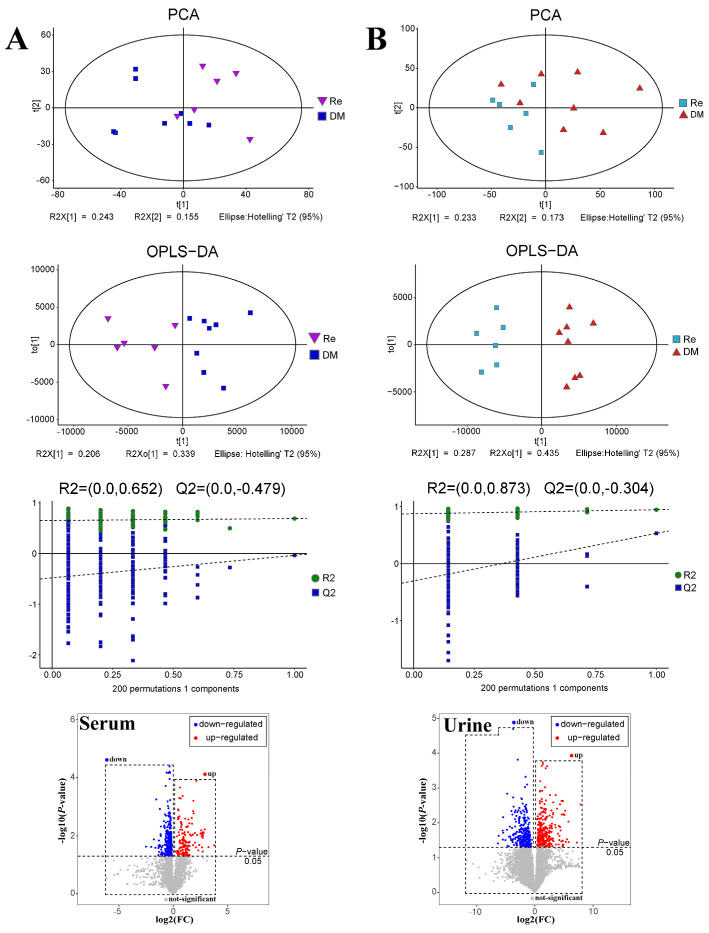
PCA analysis, OPLS-DA analysis, OPLS-DA permutation test and volcano plot of serum (**A**) and urine (**B**) samples from the ginsenoside Re compared to T2DM group, respectively.

PCA was an unsupervised multivariate analysis method used to reflect the overall distribution trend among samples. For each comparison group of serum (Figure 5A) and urine (Figure 5B), PCA score plots showed an obvious trend of separation, suggesting that the Pharmacological experiment model was established successfully, and the drug intervention changed the metabolism in rats.

A supervised OPLS-DA model was established to further screen the differential metabolites. The separated clusters were observed between samples of each contrast group. The OPLS-DA model parameters (R2Y, Q2) were obtained through a 7-fold cross-validation to measure the fitness and the prediction ability of the model. R2Y values in serum (R2Y = 0.69) and urine (R2Y = 0.94) were all greater than 0.5, suggesting that the model could meet the predictive ability of the data. Then, the OPLS-DA model was assessed by a permutation to verify the quality of the model, overfitting did not occur according to the results. Both PCA and OPLS-DA score plots showed an obvious separation trend in serum and urine comparison groups, indicating that ginsenoside Re treatment intervened in the metabolic profiles in T2DM rats.

Univariate statistical analysis was then carried out to visualize the fold change (FC) value and significance test of difference (*p*-value) through volcanic map analysis (Figure 5), red-dots represented significantly up-regulated metabolites, blue-dots represented significantly down-regulated metabolites, and gray-dots represented insignificantly metabolites (*p* > 0.05). There were 10 (in serum) and 12 (in urine) differential metabolites filtered out in the Re compared to the T2DM group, which are represented in Table 2.

### 2.3. KEGG Pathway Enrichment Analysis

Among the differential metabolites, two compounds (in serum) and six compounds (in urine) were identified which could be enriched in KEGG metabolic pathway (shown in Table 3). Differential metabolites are putatively identified by accurate mass measurement, MS/MS spectra, and database searching. The molecular MS error of all identified metabolites was less than 10 ppm. These differential metabolites were identified by comparing the retention time, accurate mass, and MS/MS spectra with these characteristics of the authentic standards.

**Figure 6:**
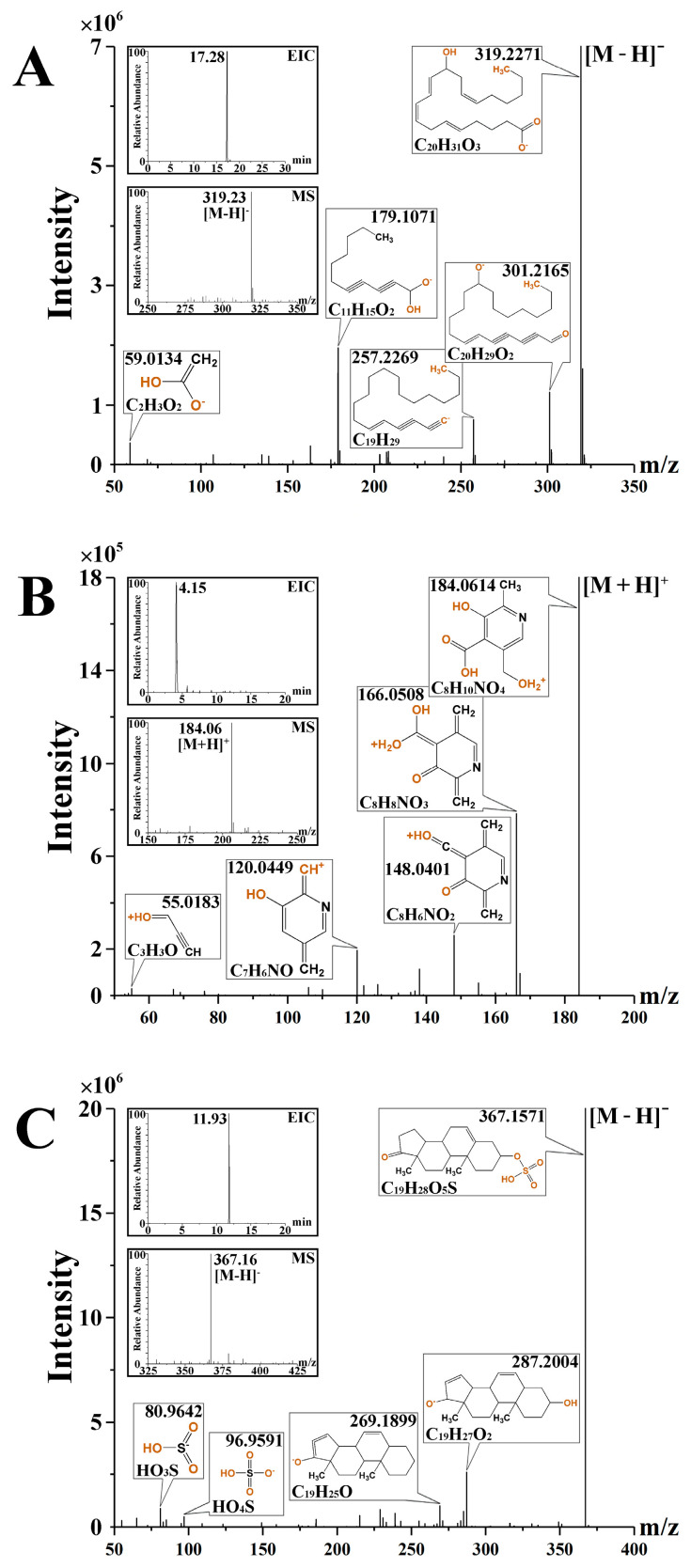
The extracted ion chromatogram, MS and MS/MS spectrum of 12(R)-HETE (**A**), 4-Pyridoxic acid (**B**) and dehydroepiandrosterone sulfate (**C**).

The three representative compounds involved in the following discussion were taken as examples to explain the metabolite identification. The extracted ion chromatogram (EIC), full scan mass spectrum, and MS/MS spectrum of compounds were shown in Figure 6. The EIC of [M − H]^−^ ion at *m/z* 319.2271 with retention time 17.28 min, the MS and MS/MS spectra in negative ion mode are shown in Figure 6A. Four major fragments ions at *m/z* 301.2165, 257.2269, 179.1071, and 59.0134 were caught in the ms^2^ spectrum, showing good accordance with that of the standard compound and spectrum in the databases. The deduced chemical structures of fragments are also shown in Figure 6A. Based on all the above information obtained, the metabolite was identified as 12(R)-HETE. [M + H]^+^ ion at *m/z* 183.0531 of 4-Pyridoxic acid (Figure 6B) in positive ion mode was detected, the main detection fragments were ions at *m/z* 166.0508, 148.0401, 120.0449, 55.0183, and the major fragments of compound dehydroepiandrosterone sulfate (Figure 6C) with an [M − H]^−^ ion of *m/z* 368.1657 were detected as ions at *m/z* 287.2004, 269.1899, 96.9591, 80.9642. The MS/MS spectra of the two identified metabolites that involved in the following discussion are also provided in Figure 6B,C. All the identified metabolites were identified based on the accurate mass measurement and MS/MS product ion analysis compared with authentic standards or databases.

Pathway enrichment analysis was performed using KEGG online database to explore the best-fit pathways and potential metabolic mechanism. Two (in serum) and six (in urine) metabolites were enriched in the KEGG pathway, respectively, as shown in Table 3.

## 3. Discussion

The differential metabolite 12(R)-HETE was detected in serum, with up-regulated expression (log_2_FC > 0). It is a 12-lipoxygenase (12-LO)-mediated arachidonic acid decomposition product, which is widely associated with the dysfunction of human and mouse pancreatic islet β-cell [23]. The level of 12(R)-HETE in patients with diabetes is controversial, some studies have shown an increase, while others have shown no change or a decrease in the level. However, it was both an important metabolite associated with diabetes [24]. Arachidonic acid (AA) had potent anti-inflammatory and anti-diabetic effects, and the levels of AA in the liver and plasma were significantly decreased in obese and T2DM rats [25]. The up-regulation showed that ginsenoside Re could activate the AA metabolic pathway and improve diabetes in the diabetic rat model. The compound was also detected during the intervention of total ginsenoside of ginseng berry in T2DM rats, indicating that ginsenoside Re was one of the pharmacodynamic substances of ginseng berry on anti-diabetes [20].

4-Pyridoxic acid was detected in urine. The expression was downregulated (log_2_FC < 0), and participated in the Vitamin B6 metabolism pathway. Vitamin B6 deficiency is common in diabetic patients [26], and studies have shown that the ability of vitamin B6 of diabetic patients to decompose into 4-Pyridoxic acid was heightened [27]. The up-regulation of 4-Pyridoxic acid was related to renal insufficiency or inflammation, and an excess of 4-pyridoxic acid was rapidly excreted in urine [28]. Diabetic nephropathy was the most common cause of end-stage kidney disease as well as renal failure [26]. The up-regulation of 4-pyridoxic acid means that ginsenoside Re could improve diabetic kidney disease.

The differential metabolite dehydroepiandrosterone sulfate (DHEA-S) was detected in urine, the expression was up-regulated (log_2_FC > 0), and participated in steroid hormone biosynthesis and bile secretion. The DHEA-S was the sulfated form of dehydroepiandrosterone (DHEA), and most DHEA was found in the form of DHEA-S, which is about 300 times higher than that of free DHEA. They were the most abundant adrenal steroids in human circulation [29]. DHEA-S could enhance insulin secretion of the pancreas and improve insulin sensitivity of muscle and liver. Moreover, it had a beneficial effect on diabetes and obesity [30]. In addition, a low concentration of DHEA-S in serum could lead to insulin resistance, while high insulin concentrations could down-regulate the concentration of DHEA-S by inhibiting promotion and producing its clearance [31]. There was a significant correlation between the onset of T2DM and a decrease in DHEA-S level, and a higher DHEA-S level in serum had a significant protective effect on the onset of T2DM in older men [32]. It has been reported that DHEA-S can up-regulate the expression of ACC1, and promote insulin secretion, which had a useful therapeutic effect on diabetes mellitus, especially for patients with insulin secretion deficiency [29]. Furthermore, the degree of diabetic nephropathy in men was inversely related to the level of DHEA-S [33]. The up-regulation of DHEA-S means that ginsenoside Re could improve its concentration and promote insulin secretion, so as to exert a part in the improvement and treatment of T2DM.

2-Arachidonylglycerol (2-AG) was detected in urine, up-regulation (log_2_FC > 0), and participated in retrograde endocannabinoid signaling. Excessive energy intake may lead to obesity, insulin resistance, and various metabolic syndrome complications, and then lead to diabetes. 2-AG could activate an endocannabinoid receptor CB_2_, the activation of CB_2_ could improve obesity and its related metabolic disorders [34]. It has been reported that 2-AG was a key signal molecule to improve insulin resistance and inflammation. 2-AG can activate AMPK by stimulating cannabinoid type-1 receptor (CB_1_) and Ca^2+^/calmodulin-dependent protein kinase β (CaMKK β), inhibit insulin resistance induced by TNF α, improve glucose uptake, and improve diabetes mellitus [35]. The up-regulation of 2-AG meant that ginsenoside Re could activate the AMPK signal pathway, against insulin resistance and improve diabetes. The metabolite Riboflavin, Allocholic acid from serum, Estrone glucuronide, Tetrahydrocortisol, and 17-β-estradiol-3-glucuronide from urine were also enriched in the KEGG pathway, as shown in Table 3.

In the recent decade, due to the expansion and application of metabolomics, the latest understanding of T2DM pathophysiology has been continuously expanded. In addition to branched-chain amino acids, phospholipid metabolism, and glutamine glutamate cycle, which were previously unknown diabetes pathways and biomarkers, vitamin B6 metabolism pathway were also reported to be associated with diabetes [21], which was also consistent with the screening results in this study. The appearance of metabolomics helped realize the discovery of these pathways and biomarkers. Moreover, it has been reported that the anti-diabetic mechanism of ginseng may be related to its regulation of insulin secretion, improvement of glucose uptake, and oxidative stress [6], and the mechanism of ginsenoside Re’s anti-diabetic effect was basically consistent with previous reports.

According to the results of the hypoglycemic experiment, ginsenoside Re could reduce the fasting blood glucose of T2DM rats. After 30 days of intervention, the hypoglycemic rate could reach 21.97%, but the hypoglycemic effect was lower than that of ginseng berry total ginsenoside extract (27.35%) [20]. The pharmacological effects of traditional medicinal herbs such as ginseng had the characteristics of multi-component and multi-target, that is, the pharmacological effects of herbal medicines were usually the result of the joint action of multiple active ingredients on different biological target molecules. Although the monomer drugs were better than traditional herbal medicines in quality control, and ginsenoside Re was also the highest monomer saponin contented in ginseng berry, from the research results, it cannot replace the total ginsenosides of *Panax ginseng* berry in lowering fasting blood glucose and treating T2DM.

## 4. Materials and Methods

### 4.1. Material and Reagents

Ginsenoside Re (C_48_H_82_O_18_, purity ≥98%) was purchased from the National Institutes for Food and Drug Control (Beijing, China). Streptozotocin (STZ) was obtained from Sigma Chemical Corp. (St. Louis, MO, USA). HPLC-grade acetonitrile and formic acid were obtained from Fisher Scientific (Waltham, MA, USA). Ultrapure water was purified using the Millipore Milli-Q system (Bedford, MA, USA). Rat serum triglyceride (TG) assay kit, total cholesterol (TC) assay kit, high/low-density lipoprotein cholesterol (HDL-C/LDL-C) assay kit, non-esterified free fatty acids (NEFA) assay kit, urea nitrogen (BUN) assay kit, uric acid (UA) test kit, superoxide dismutase (SOD) assay kit, malondialdehyde (MDA) assay kit, catalase (CAT) assay kit, and glucose assay kit were purchased from Nanjing Jiancheng Biotech. Corp. (Nanjing, China). 4-Pyridoxic acid was purchased from Sigma-Aldrich Corp. (St. Louis, MO, USA), 12(R)-HETE was purchased from Cayman Chemical (Ann Arbor, MI, USA), 2-Arachidonylglycerol was purchased from Shanghai Fusheng biotech. Corp. (Shanghai, China), and dehydroepiandrosterone sulfate was purchased from Shanghai Huicheng biotech. Corp. (Shanghai, China).

### 4.2. Animal Experiments

30 seven-week-old male Wistar rats (190 ± 10 g) were obtained from the Experimental Animal Center of Jilin University (Jilin, China). The animal experiment was approved by the Institutional Animal Care and Use Committee (IACUC) of the Changchun University of Chinese Medicine; permit number: CPCCUCM IACUC 2019-008. Animals were reproduced in an SPF barrier system with standard environmental parameters: constant temperature of 21–25 °C, relative humidity of 60% ± 20%, and 12 h light–dark (6:00 a.m.–6:00 p.m.) cycle and received food and water freely. After 7 days of acclimatization, 8 rats were randomly divided into healthy control (CON) group and fed a normal diet. The others were fed a high-fat and high-sugar diet (HFD), including 10% lard oil, 20% saccharose, 2.5% cholesterol, 1% sodium cholate, and 66.5% standard rat diet, and 8 weeks later, STZ (dissolved in sodium citrate buffer, 0.1 mol/L, pH 4.3–4.5) was intraperitoneally injected into rats at a dose of 40 mg/kg b.w. and the rats in the CON group were only injected with sodium citrate buffer as parallel control. Seven days later, after fasting for 12 h, the tail-vein blood glucose was measured. The rats with blood glucose ≥7.8 mmol·L^−1^, polyuria, and polydipsia were successfully established as T2DM model.

Twenty-two T2DM rats were randomly divided into three groups. Among them, 6 rats in the Re group continued to feed with an HFD diet and were given ginsenoside Re (dissolved in saline) at a dose of 25 mg·kg^−1^ (b.w.) once a day by gastric irrigation. Eight rats in the MET group were also fed an HFD diet and given metformin (dissolved in saline) at a dose of 100 mg·kg^−1^ (b.w.) once a day. In the T2DM group, 8 rats were fed an HFD diet and only saline was given by gastric irrigation. The CON group continued to be fed with a normal diet and was also given saline in parallel. The blood glucose level of rats was measured every 7 days (after fasting for 12 h). The weight of rats and the food and water intake in 24 h were measured every 4 days.

### 4.3. Sample Collection and Preparation

#### 4.3.1. Urine Samples

The intervention lasted for 30 days, at the endpoint of treatment, rats in each group were placed in a metabolism cage (1 rat per cage). Twenty-four-hour urine samples were collected in tubes on ice from 4 groups. Then, urine samples were centrifuged at 8000 rpm at 4 °C for 15 min, and the supernatant was divided into 250 μL/portion at 4 °C and stored at −80 °C until analysis. The urine samples were thawed at 4 °C before analysis, after thawing, 250 μL of urine was exactly aspirated, added 750 μL methanol (4 °C), mixed by vortex for 30 s, and then centrifuged at 12,000 rpm at 4 °C for 15 min. The centrifuged supernatant was sucked into a new tube and evaporated under nitrogen. Then, the dried sample was redissolved with 1 mL ultra-pure water (4 °C), centrifuged at 4 °C and 12,000 rpm for 15 min, and the supernatant was filtered by 0.22 μm membrane and analyzed by UHPLC-MS. All operations were performed on ice.

#### 4.3.2. Serum Samples

To collect serum samples, the rats were fasted (12 h, overnight) and anesthetized by intraperitoneal injection of 10% chloral hydrate (3 mL/kg body weight). Blood samples were vacuum-collected from the abdominal aorta. Then, the blood sample was placed at room temperature for 60 min and centrifuged at 3000 *g* at 4 °C for 15 min. The supernatant was separated from the blood sample and the serum sample was obtained. The serum was packed into 300 μL/portion and immediately frozen in liquid nitrogen, then stored at −80 °C until analysis. One part was used for determining rat serum biochemical parameters according to the commercial kit instructions, and the other was used for UHPLC-MS detection. Before the analysis, the serum was thawed at a temperature of 4 °C. After thawing, 250 μL of serum was accurately aspirated and added to 750 μL of methanol solution (4 °C) in order to precipitate the proteins, which was mixed by vortex (30 s) and centrifuged at 12,000 rpm for 15 min at 4 °C. After the supernatant was transferred into a new tube and dried by a nitrogen-blowing instrument, 500 μL of methanol was again added, vortexed, and centrifuged at 12,000 rpm for 10 min. After the supernatant was collected again and dried, the solid sample was dissolved in 1 mL of ultra-pure water and centrifuged at 12,000 rpm at 4 °C for 15 min. Then, the supernatant was filtered by a 0.22 μm membrane filter, and then analyzed by UHPLC-MS. All the processing was performed on ice.

#### 4.3.3. Quality Control Sample

In order to test the stability and reproducibility of UHPLC-MS, quality control (QC) samples were prepared. Among all the samples, small parts of the same volume were removed, mixed, and treated according to the pretreatment method of the samples to obtain the QC samples of serum and urine samples, respectively. Before the experiment, the QC sample was detected six times to balance the analysis system, then QC samples and blank samples (75% acetonitrile in water) were detected once between every six real samples in the sequence.

### 4.4. UHPLC-MS Analyses

Serum and urine metabolomic profiling was analyzed by the UHPLC Q-Exactive Orbitrap MS system, and chromatographic separation was detected on an UltiMate 3000 system (Sunnyvale, CA, USA) with C_18_ column (50 × 2.1 mm, 1.9 μm, Thermo Fisher Scientific) maintained at 35 °C. Mobile phase A was 0.1% formic acid in water and mobile phase B was 100% acetonitrile with a flow rate of 0.3 mL/min. The proportion of acetonitrile (B) gradient was used for serum as follows: 0–1 (min): 3%, 1–16 (min): 3–70%, 16–18 (min): 70%, 18–25 (min): 70–90%, 25–26 (min): 90–100%, 26–30 (min): 100%. Another 20 min elution gradient of acetonitrile (B) for urine analysis was carried out as follows: 0–5 (min): 5–20%, 5–10 (min): 20–35%, 10–15 (min): 35–98%, 15–16 (min): 98–100%, 16–20 (min): 100%. The injection volume was 2.0 μL, and samples were kept at a constant temperature of 4 °C.

Mass spectrometric detection was implemented on a Q-Exactive Orbitrap MS (Thermo, CA, USA) equipped with electrospray ionization (ESI) source in both positive and negative ion modes. The scanning range was *m/z* 66.7~1000. The ionization source parameters were set as follows: sheath gas flow was 6.125Mpa, aux gas flow was 2.625 Mpa, and sweep gas flow was 0.175 Mpa. The capillary temperature was 320 °C, the auxiliary gas heater temperature was 350 °C, and the capillary voltage was 3.5 kv.

Scanning mode was Full MS with dd-MS^2^, Top N = 8. The resolution was 70,000 in Full MS mode, and 17,500 in data-dependent MS^2^ mode, respectively. The normalized collision energy (NCE) was 10%, 20%, and 40%. The software for recording and analyzing data were Dionex^TM^, Chromeleon^TM^ 6.8, and Xcalibur software Version 2.2.42 (Thermo Fisher Scientific, San Jose, CA, USA).

### 4.5. Multivariate Data Processing and Analysis

Serum and urine raw mass data from the UHPLC-MS was imported into the Progenesis QI v2.3 software (Nonlinear Dynamics, Newcastle, UK), for peak alignment, deconvolution, correction retention time, normalization, and integration. The software could carry out normalization processing of the data based on QC samples. During data collection, insert and collect the same QC sample every six samples, which could be used to simulate signal changes during data acquisition, and then, normalized the data through QI v2.3 software. More than 50% of the metabolites with missing measured values were not used for a follow-up analysis in the extracted ion features. Both the positive and negative ion data were merged into a data matrix and then saved as an excel file.

The unsupervised PCA (principal components analysis) and the supervised OPLS-DA (orthogonal partial least squares discriminant analysis) were performed on R programming language v3.6.2 (Ropls package was used for multivariate statistics). The robustness of the OPLS-DA model was evaluated by 7-fold cross-validation and 200 times response permutation testing (RPT). Univariate statistical analysis was used for basic significance statistics by Stats package (ggplot2 package was used for plotting). The differential metabolite was selected on the basis of two criteria: (a) the VIP value from the OPLS-DA model should be satisfied with VIP > 1 and (b) the differences from the Univariate statistical analysis (Student’s *t*-test) should be satisfied with *p* < 0.05. Data were shown as the mean ± standard deviations (SD).

### 4.6. Differential Metabolites Identification and Pathway Analysis

The identification of potential metabolites was based on the product ion mass spectra and MS/MS fragment ion and suited with the structural message in the databases, such as HMDB (http://www.hmdb.ca/) (accessed on 7 December 2019), METLIN (https://metlin.scripps.edu/) (accessed on 7 December 2019), and Lipidmaps (http://www.lipidmaps.org/) (accessed on 7 December 2019). The metabolic pathway analysis of the differential metabolites was performed by the online database KEGG (http://www.kegg.jp/) (accessed on 7 December 2019).

## 5. Conclusions

UHPLC-MS was used to study the anti-diabetic effect of ginsenoside Re on T2DM rats induced by a high-fat diet and streptozotocin. The biochemical and pharmacological indexes showed that ginsenoside Re could significantly reduce the level of fasting blood glucose and serum lipid, improving oxidative stress and renal function in T2DM rats, which proved that ginsenoside Re could improve T2DM. There were 10 (in serum) and 12 (in urine) differential metabolites that were screened in the ginsenoside Re vs T2DM group. Among the differential metabolites, two compounds (in serum) and six compounds (in urine) were identified and analyzed which could be enriched in the KEGG metabolic pathway. The differential metabolites were mainly involved in arachidonic acid metabolism, Vitamin B6 metabolism, steroid hormone biosynthesis, and the bile secretion metabolic pathway. Moreover, ginsenoside Re could promote insulin secretion, stimulate cannabinoid type 1 receptor (CB_1_) and CaMKK β to activate the AMPK signaling pathway, inhibit insulin resistance induced by TNF-α, and improve blood glucose uptake and diabetic nephropathy, so as to exert the goal of anti-diabetic.

## Figures and Tables

**Table 1:** Biochemical parameters of rats in different groups after treatment for 30 days.

Items	CON	T2DM	MET	Re
Triglyceride TG (mmol/L)	0.58 ± 0.14 **	1.65 ± 0.34	0.91 ± 0.55 **	1.11 ± 0.44 *
Total cholesterol TC (mmol/L)	2.39 ± 0.27 **	4.86 ± 1.21	3.52 ± 0.92 *	3.80 ± 1.05
High-density lipoprotein cholesterol HDL-C (mmol/L)	1.28 ± 0.24	1.11 ± 0.21	1.29 ± 0.23	1.18 ± 0.25
Low-density lipoprotein cholesterol LDL-C (mmol/L)	0.47 ± 0.29 **	1.99 ± 0.77	0.93 ± 0.43 **	1.09 ± 0.52 *
Non-esterified Free fatty acids NEFA (μ mol/L)	0.38 ± 0.15 *	0.69 ± 0.34	0.42 ± 0.13	0.60 ± 0.21
Urea nitrogen BUN (mmol/L)	4.00 ± 0.86 **	8.75 ± 1.62	6.13 ± 1.84 **	6.61 ± 1.33 *
Uric acid UA (μ mol/L)	135.59 ± 31.43 *	264.37 ± 134.67	160.89 ± 34.31	182.59 ± 98.14
Malondialdehyde MDA (nmol/mL)	5.29 ± 0.70 **	7.42 ± 1.29	5.94 ± 0.74 *	6.06 ± 0.69 *
Super oxide dismutase SOD (U/mL)	195.94 ± 10.19 **	161.02 ± 10.16	180.70 ± 14.74 **	175.07 ± 10.83 *
Catalase CAT (U/mL)	31.91 ± 0.83 **	20.02 ± 5.33	26.72 ± 6.60 *	25.86 ± 8.30

Data are expressed as the mean ± SD. * *p* < 0.05 vs. T2DM group; ** *p* < 0.01 vs. T2DM group.

**Table 2:** Identification results of differential metabolites in T2DM vs. ginsenoside Re group in serum and urine.

Source	No.	Mode	Metabolite	Formula	Adduct	tR/min	Measured m/z	MS/MS	Error (ppm)	Log2(FC)	VIP	*p*-Value
Serum	1	Pos	Riboflavin reduced	C_15_H_16_N_4_O_6_	[M + Na]^+^	4.79	371.0960	158.0712, 61.0282	−0.7	−0.28	4.01	0.00007
	2	Pos	Lucidenic acid A	C_27_H_38_O_6_	[M + H]^+^	8.79	459.2744	441.2643, 423.2537, 149.0968	1.3	−0.14	5.56	0.04836
	3	Neg	PG(22:6(4Z,7Z,10Z,13Z,16Z,19Z)/0:0)	C_28_H_45_O_9_P	[M − H_2_O − H]^−^	9.04	537.2646	268.8006, 152.8823, 92.9266	4.2	−0.27	1.35	0.01925
	4	Neg	LysoPE(0:0/24:0)	C_29_H_60_NO_7_P	[M − H]^−^	9.76	564.4042	349.3481, 196.0385, 78.9596	2.1	−0.51	1.88	0.02859
	5	Neg	PA (13:0/18:3(6Z,9Z,12Z))	C_34_H_61_O_8_P	[M − H]^−^	9.78	627.4042	462.7301, 356.8575	1.7	−0.46	1.16	0.02889
	6	Neg	PS (14:1(9Z)/20:1(11Z))	C_40_H_74_NO_10_P	[M − H_2_O − H]^−^	10.23	740.4875	551.6436, 468.4839, 450.4845	0.5	−0.29	1.05	0.00818
	7	Neg	Allocholic acid	C_24_H_40_O_5_	[M − H]^−^	13.95	407.2785	278.8336, 176.9123	−4.4	2.61	3.70	0.01039
	8	Neg	1-(11Z,14Z-eicosadienoyl)-glycero-3-phosphate	C_23_H_43_O_7_P	[M − H_2_O − H]^−^	13.97	443.2547	152.9943, 96.9687, 78.9586	−4.6	2.70	1.14	0.00798
	9	Neg	12(R)-HETE	C_20_H_32_O_3_	[M − H]^−^	17.28	319.2271	301.2165, 179.1071, 59.0134	−2.5	1.02	1.51	0.01490
	10	Neg	PG (22:0/21:0)	C_49_H_97_O_10_P	[M − H_2_O − H]^−^	22.41	857.6682	326.8663, 232.5573	4.6	−1.15	1.73	0.00989
Urine	1	Pos	Kuwanon E	C_25_H_28_O_6_	[M + H]^+^	1.91	425.1970	407.1866, 153.0193, 69.0707	2.8	1.51	1.17	0.01415
	2	Pos	4-Pyridoxic acid	C_8_H_9_NO_4_	[M + H]^+^	4.15	184.0614	166.0508, 148.0401, 120.0449	1.6	−1.99	2.99	0.04283
	3	Pos	(3R,7R)-1,3,7-Octanetriol	C_8_H_18_O_3_	[M + Na]^+^	7.71	185.1146	99.0805, 69.0701	−1.1	−2.56	1.56	0.01692
	4	Pos	Lucidenic acid A	C_27_H_38_O_6_	[M + H]^+^	8.79	459.2744	411.2539, 379.2276, 83.0863	1.3	0.58	8.26	0.00762
	5	Neg	Estrone glucuronide	C_24_H_30_O_8_	[M − H]^−^	7.60	445.1879	401.1975, 269.1551, 87.0087	2.4	1.64	1.29	0.03203
	6	Neg	17-β-estradiol-3-glucuronide	C_24_H_32_O_8_	[M − H_2_O − H]^−^	9.40	429.1929	271.1708, 89.0246	2.2	1.48	1.89	0.04473
	7	Neg	17-β-estradiol glucuronide	C_24_H_32_O_8_	[M − H_2_O − H]^−^	9.96	429.1930	271.1709, 255.1392, 117.0191	2.4	3.20	2.26	0.00515
	8	Neg	Dehydroepiandrosterone sulfate	C_19_H_28_O_5_S	[M − H]^−^	11.93	367.1571	287.2004, 269.1899, 96.9591	−2.6	1.79	1.08	0.00096
	9	Neg	2-Arachidonylglycerol	C_23_H_38_O_4_	[M + FA − H]^−^	12.31	423.2735	162.0226, 96.9635	−4.5	4.45	1.43	0.01662
	10	Neg	Tetrahydrocortisol	C_21_H_34_O_5_	[M − H]^−^	12.91	365.2320	347.2211, 335.2216, 289.2163	−3.7	2.47	2.65	0.01919
	11	Neg	Armillaripin	C_24_H_30_O_6_	[M − H]^−^	13.40	413.1986	249.1506, 231.1396, 137.0611	4.1	4.82	1.00	0.04689
	12	Neg	1β-Hydroxycholic acid	C_24_H_40_O_6_	[M − H_2_O − H]^−^	13.48	405.2629	387.2530, 361.2739, 59.0131	−4.2	4.78	1.21	0.03268

**Table 3:** Enriched KEGG pathway of metabolites from Ginsenoside Re vs. T2DM group.

Origin	No.	Metabolite	Compound ID	KEGG	Trend	Annotation
Serum	1	12(R)-HETE	HMDB0062287	C14822	↑	Aldosterone synthesis and secretion, and Arachidonic acid metabolism
	2	Riboflavin reduced	HMDB0001557	C01007	↓	Riboflavin metabolism
Urine	1	4-Pyridoxic acid	HMDB0000017	C00847	↓	Vitamin B6 metabolism
	2	Dehydroepiandrosterone sulfate	HMDB0001032	C04555	↑	Bile secretion, and Steroid hormone biosynthesis
	3	Estrone glucuronide	HMDB0004483	C11133	↑	Steroid hormone biosynthesis
	4	2-Arachidonylglycerol	HMDB0004666	C13856	↑	Retrograde endocannabinoid signaling, Thermogenesis, and Neuroactive ligand–receptor interaction
	5	Tetrahydrocortisol	LMST02030143	C05472	↑	Steroid hormone biosynthesis
	6	17-β-estradiol-3-glucuronide	LMST05010058	C03033	↑	Bile secretion, and Pentose and glucuronate interconversions

## Data Availability

All the data used in this study are available within this article. Further inquiries can be directed to the authors.

## Sample Availability

Samples of the compounds are not available from the authors.
